# Genomic selection in tree breeding: testing accuracy of prediction models including dominance effect

**DOI:** 10.1186/1753-6561-5-S7-O13

**Published:** 2011-09-13

**Authors:** Marie Denis, Jean-Marc Bouvet

**Affiliations:** 1 CIRAD - Research unit 108 "Breeding and adaptation of tropical and Mediterranean plants", International campus of Baillarguet TA A-39/C, 34398 Montpellier , Cedex 5, France

## Background

The concept of Marker Assisted Selection (MAS) is rapidly evolving in animal and plant breeding. With the advent of high throughput molecular technology, numerous molecular markers distributed throughout the whole genome can be produced to characterize many genetic entries involving new perspectives in methodology of selection. An important research activity has begun in the animal world given the first theoretical framework for a methodology called genomic selection (GS) [1]. Several statistical approaches have been proposed for the prediction of genomic breeding values and numerous results are available that validates the interest of this method in animal breeding. In plants the GS is still limited to very advanced model species involved in genetic improvement and especially from scenario-based simulation [2,3].

In tree breeding the GS could significantly reduce the cost of genetic improvement schemes by limiting the size and number of field experiments; and facilitating the early selection at the nursery stage [4]. If most of the studies on GS have addressed the prediction of breeding value, taking into account the gene additive effects, there is still a lack of analyses dealing with the total genetic value (genotypic value) including both additive and dominance effects. This aspect is important in plant and especially in tree breeding where the goal of some programs is the production of clones or elite families. The aim of this study is to investigate the performance of GS in the context of tree breeding when the selection is based on genotypic value. The proposed approach allows taking into account both additive and dominance effect [5] for each marker in the statistical model. Six scenarios are simulated to test the reliability of the GS in the frame of recurrent selection scheme.

## Methods

### Simulation

The data used to evaluate the accuracy of the model have been simulated using HaploSim package in R software [6].

Firstly, populations were simulated for 1000 generations at an effective size of 100 to reach a mutation-drift balance. Fifty parent trees were then selected to start a breeding scheme that was conducted during two generations. At each generation, a progeny test was implemented using a factorial mating design. The fifty percent parents were selected and crossed using circular design to constitute the following generation. At each generation, 670 individuals issued from the mating of 16 females and 34 males were evaluated for clonal selection. The 670 individuals were genotyped for 400 SNP markers equally-spaced across one chromosome of one Morgan corresponding to an efficient marker density.

The broad sense heritability HÂ² was equal to 0.3. A gamma distribution was used to sample the 44 QTL effects.

The additive (breeding), dominance and genotypic values were simulated for each individual. The ratio of dominance to additive variance was equal to 0.1, 0.5 and 1. Six scenarios were evaluated for predicting the genotypic value: three different ratios and two different QTL distributions (high proportion with small or medium effects).

### Analysis model

Genomic selection consists in following steps: (i) estimation of the effects of all markers in a 'training data set', where the individuals are phenotyped and genotyped; (ii) prediction of the genetic values of other 'evaluation' individuals by combining their marker genotypes with the estimates obtained in step (i) .

A Bayesian implementation of the Lasso method with BLR package in R [7,8] was used to estimate the substitution and dominance effects for each of the 400 SNP. This method allows predicting the genotypic value using all markers simultaneously with different variances for each marker effect. We evaluated the performance of the statistical model with and without dominance effects. The training and validation set corresponded, respectively, to the first and second generation containing each 670 individuals. The criterion to compare the different scenarios was the accuracy calculated as the correlation between true and predicted genotypic value. Each simulated data set and analysis was replicated 30 times.

## Results

The accuracy of two models decreases when the ratio of dominance to additive variance increases whatever the QTL distribution (table 1). In all scenarios GS is superior to basic phenotypic selection. In addition, the model with dominance effects shows a higher accuracy, especially when the variance ratio increases. For the second generation the same trend is observed but the model with dominance effects is more accurate when the ratio of variances is greater than 0.5. A lower accuracy is observed in the second generation; it can be attributed to the low linkage disequilibrium of this breeding population two generations after selection in simulated wild population.

**Table 1:** Accuracy (se) of GS in the first and second generation without and with dominance effects for six different scenarios and accuracy of phenotypic selection (30 replicates)

ratio	QTL distribution: small effects			QTL distribution: medium effect		
**Dominance variance/additivevariance**	Phenotypic selection	Model without dominance	Model with dominance	Phenotypic selection	Model without dominance	Model with dominance

**First generation**						

0.1	0.52(0.03)	0.80(0.03)	0.81 (0.03)	0.53(0.03)	0.79 (0.03)	0.80(0.03)
0.5	0.51(0.03)	0.70 (0.05)	0.73 (0.04)	0.53(0.04)	0.68 (0.04)	0.73(0.04)
1	0.51(0.03)	0.61(0.06)	0.69(0.05)	0.53(0.03)	0.64(0.04)	0.71(0.04)

**Second generation**						

0.1	NO	0.63(0.13)	0.63(0.13)	NO	0.62(0.08)	0.62(0.08)
0.5	NO	0.53(0.13)	0.55(0.12)	NO	0.53(0.11)	0.54(0.10)
1	NO	0.42(0.13)	0.48(0.13)	NO	0.42(0.10)	0.47(0.9)

NO: not observed

## Conclusions

The model including dominance effects is more accurate to predict the genotypic value especially when the dominance-additive variance ratio increases. These results are particularly interesting for tree improvement in hybrid populations where dominance effects are marked and clonal varieties are produced (eucalyptus, poplar, for example).

## References

[B1] Meuwissen THE Hayes BJ Goddard ME Prediction of total genetic value using genome-wide dense marker maps Genetics 2001 157 1819 1829 11290733 10.1093/genetics/157.4.1819 PMC1461589

[B2] Bernardo R Yu JM Prospects for genomewide selection for quantitative traits in maize Crop Sci 2007 47 1082 1090 10.2135/cropsci2006.11.0690

[B3] Heffner EL Sorrells ME Jannink JL Genomic Selection for Crop Improvement Crop Sci 2009 49 1 12 10.2135/cropsci2008.08.0512

[B4] Grattapaglia D Resende MDV Genomic selection in forest tree breeding Tree Genetics and Genomes 2011 7 241 255 10.1007/s11295-010-0328-4

[B5] Toro MA Varona L A note on mate allocation for dominance handling in genomic selection Genet Sel Evo 2010 42 1 33 10.1186/1297-9686-42-1 PMC2928189 20699012

[B6] Coster A Bastiaansen J HaploSim: HaploSim 2009

[B7] De los Campos G Naya H Gianola D Crossa J Legarra A Manfredi E Weigel K Cotes JM Predicting quantitative traits with regression models for dense molecular markers and pedigrees Genetics 2009 182 375 385 10.1534/genetics.109.101501 19293140 PMC2674834

[B8] De los Campos G Pérez P BLR: Bayesian Linear Regression 2010 R package version 1.1 10.1007/978-1-62703-447-0_12 23756896

